# Establishment of an *ex vivo* vascular tissue model to investigate bunyavirus-triggered vascular endothelial injury

**DOI:** 10.1128/mbio.01933-26

**Published:** 2026-09-25

**Authors:** Zihan Guan, Xi Chen, Yujie Fang, Xiangtao Zhu, Shufen Li, Ke Peng

**Affiliations:** 1 State Key Laboratory of Virology and Biosafety, Center for Antiviral Research, Center for Biosafety Mega-Science, Wuhan Institute of Virology, Chinese Academy of Sciences 74614, Wuhan, Hubei, China; 2 University of Chinese Academy of Sciences 74519, Beijing, China; 3 Department of Vascular Surgery, Wuhan No. 1 Hospital 593237 , Wuhan, Hubei, People’s Republic of China; Huazhong Agricultural University, Wuhan, Hubei, China

**Keywords:** bunyavirus, human vascular tissue, *ex vivo *model, endothelial cell damage, antiviral compound

## Abstract

Severe fever with thrombocytopenia syndrome, an emerging infectious disease caused by the tick-borne bunyavirus severe fever with thrombocytopenia syndrome virus (SFTSV), is associated with a high clinical fatality rate of 12%–50%. Vascular endothelial injury triggered by SFTSV infection has been linked to severe disease progression and mortality. Currently, a suitable model to investigate the underlying mechanisms of SFTSV-induced vascular damage is lacking. Here, we show that the *ex vivo* human vascular tissue can be used as a model for studying bunyavirus-triggered endothelial injury. Both SFTSV and Rift Valley fever virus (RVFV) can successfully infect *ex vivo* human vascular tissue, as evidenced by detection of viral nucleoprotein and viral amplification in the tissue. Histopathological analysis, TUNEL staining, and endothelial injury-associated transcriptional changes revealed irregular venous tissue structure, luminal endothelial disruption, and apoptosis following infection. This model enables evaluation of antiviral compound efficacy, demonstrating that niclosamide or 4′-fluorouridine significantly inhibits SFTSV and RVFV replication in *ex vivo* vascular tissues. These findings establish the *ex vivo* human vascular tissue as a valuable tool for investigating bunyavirus-induced vascular pathogenesis and for evaluating antiviral therapeutics.

**IMPORTANCE:**

Severe fever with thrombocytopenia syndrome virus and other pathogenic bunyaviruses cause severe human diseases that are frequently associated with vascular dysfunction. However, the mechanisms underlying virus-induced vascular injury remain poorly defined due to the lack of physiologically relevant human tissue models. Here, we establish an *ex vivo* human vascular tissue model that supports productive bunyavirus infection and enables quantitative assessment of endothelial injury, tissue damage, and antiviral efficacy. Using this model, we demonstrate that bunyavirus infection triggers endothelial disruption and that antiviral compounds can suppress viral replication and alleviate infection-associated tissue injury. This model provides a valuable experimental system for studying virus-vascular interactions and can support antiviral development against emerging bunyaviruses.

## INTRODUCTION

Severe fever with thrombocytopenia syndrome (SFTS) is an emerging tick-borne infectious disease caused by the severe fever with thrombocytopenia syndrome virus (SFTSV), also known as Dabie bandavirus, which is classified within the species *Bandavirus dabieense* (1, 2). This virus belongs to the genus *Bandavirus* within the *Phenuiviridae* family of the *Hareavirales* order (3). The SFTSV genome consists of three negative-sense RNA segments encoding four proteins, including nonstructural protein S, RNA-dependent RNA polymerase, nucleoprotein (NP), and glycoprotein precursor (Gn and Gc). Clinically, SFTS manifests as high fever, vomiting, thrombocytopenia, leukocytopenia, encephalitis, hemorrhage, and multi-organ failure, with a mortality rate ranging from 12% to 50% (4–6).

Since its identification in China in 2009, SFTSV infection has been reported in multiple Asian countries, reflecting its expanding geographic distribution and global health concern (1, 7–12). SFTSV belongs to an expanding group of clinically relevant tick-borne bunyaviruses, which includes related bandaviruses such as Heartland virus (HRTV) and Guertu virus (GTV), as well as other hemorrhagic bunyaviruses such as Rift Valley fever virus (RVFV). These emerging viruses represent significant public health threats due to their ability to cause severe systemic disease and limited availability of effective antiviral therapies. Due to its high fatality rate and expanding geographic distribution, SFTSV represents an important global public health concern.

Currently, the pathogenic mechanism of SFTSV infection remains elusive. Clinical observations have linked hemorrhage, disseminated intravascular coagulation, and multi-organ dysfunction to disease severity and mortality (13). These pathophysiological features resemble those observed in viral hemorrhagic fevers (VHFs) caused by other hemorrhagic fever viruses. Vascular endothelial injury, characterized by increased vascular permeability and endothelial barrier disruption, has been implicated in VHF pathogenesis (14, 15). SFTSV-induced endothelial damage has been reported to contribute to vascular leakage, hemorrhage, and severe disease development in infected patients (16). Notably, similar mechanisms have been proposed for other hemorrhagic bunyaviruses such as RVFV (17). However, a suitable model system for studying virus-induced vascular endothelial injury is currently lacking.

In this study, we established an *ex vivo* human vascular tissue explant model to investigate bunyavirus-induced vascular injury. Human vascular tissues collected from discarded surgical specimens supported productive infection by SFTSV and RVFV and exhibited measurable infection-associated pathological alterations, including endothelial injury, increased cell death, and disruption of vascular homeostasis. This system further enabled evaluation of antiviral compounds against bunyaviruses. Using this model, we identified that niclosamide and 4′-fluorouridine (4′-Flu) exhibit potent inhibitory effects against bunyaviruses. We further show that niclosamide inhibits SFTSV internalization through inhibition of autophagy. The *ex vivo* vascular tissue model provides a physiologically relevant system for mechanistic investigation of vascular endothelial injury induced by bunyavirus infection and for the development of antiviral therapeutics.

## RESULTS

### Bunyaviruses establish infection in *ex vivo* human vascular tissue

**Fig 1:**
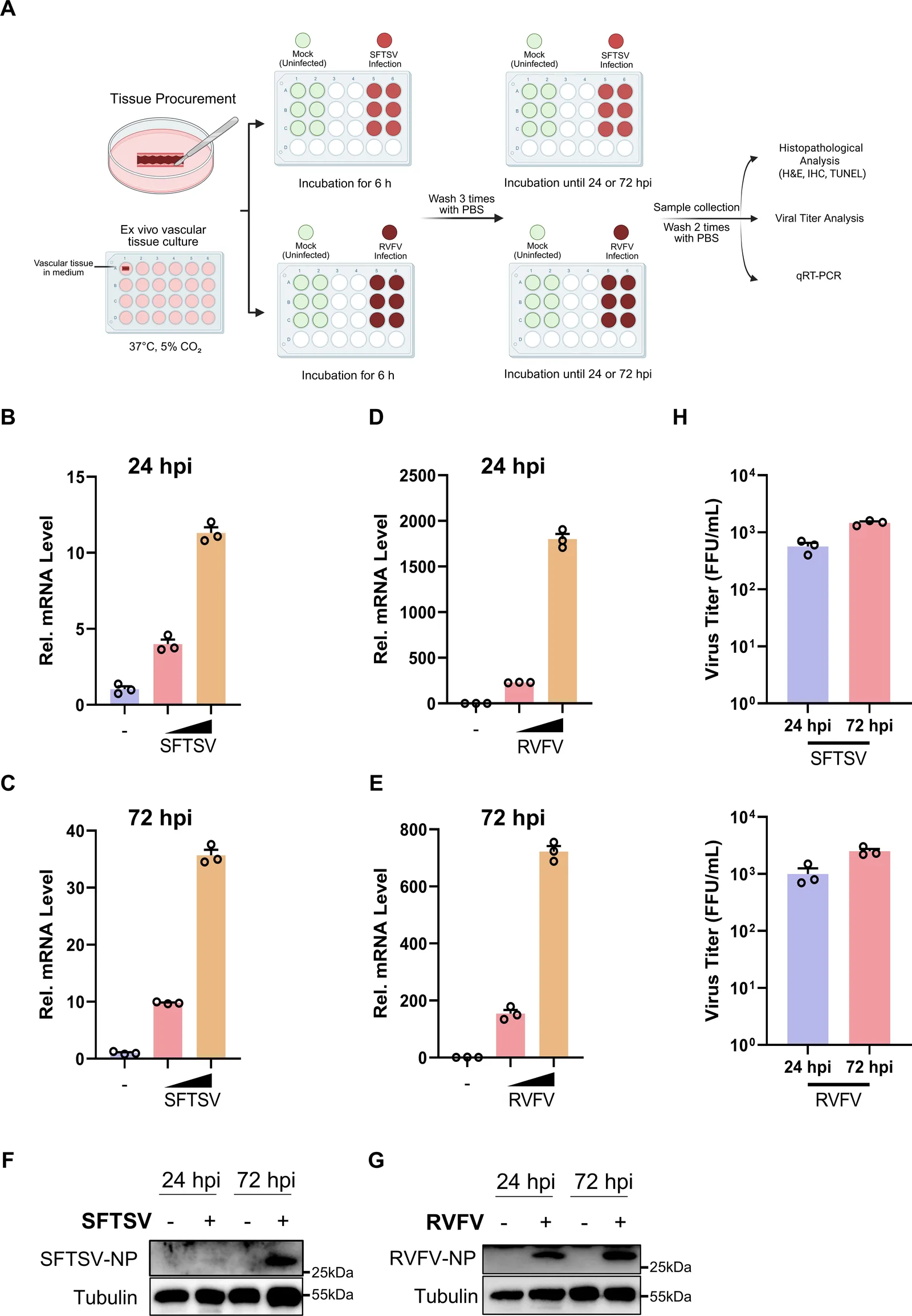
Bunyaviruses efficiently infect *ex vivo* vascular tissue. (**A**) Schematic representation of the experimental workflow for the *ex vivo* human vascular tissue model. *Ex vivo* vascular tissues were pre-cultured at 37°C with 5% CO_2_ for 6 h and then subjected to mock treatment or infection with SFTSV or RVFV. Following 6 h of inoculation, tissues were washed three times with PBS and further cultured until 24 or 72 h post-infection (hpi). Samples were subsequently collected for histopathological analysis (hematoxylin and eosin, immunohistochemistry (IHC), and TUNEL), viral titer determination, and qRT-PCR. Green wells represent mock-treated tissues, light red wells represent SFTSV-infected tissues, and dark red wells represent RVFV-infected tissues. (**B through E**) *Ex vivo* vascular tissues were inoculated with SFTSV or RVFV (1 × 10^6^ or 4 × 10^6^ focus-forming units [FFU] per sample) for 6 h. Infected *ex vivo* vascular tissues were collected at 24 and 72 hpi. Viral RNA levels were quantified by qRT-PCR and normalized to 18S rRNA. (**F and G**) *Ex vivo* vascular tissues were inoculated with SFTSV or RVFV at 4 × 10^6^ FFU per sample for 6 h. Infected *ex vivo* vascular tissues were collected at 24 and 72 hpi for western blot analysis. (**H**) *Ex vivo* vascular tissues were inoculated with SFTSV or RVFV at 4 × 10^6^ FFU per sample for 6 h. At 24 and 72 hpi, culture supernatants were collected, and the infectious virus titers (FFU/mL) were determined by a focus-forming assay. Data are presented as means ± SEM (*n* = 3, from three independent tissue donors) in panels **B–E** and **H**.

To investigate the mechanisms underlying bunyavirus-induced vascular injury, we developed a human *ex vivo* vascular tissue model using vascular tissue obtained from patients undergoing lower-limb varicose vein surgery. The tissue procurement was conducted in accordance with bioethical guidelines and coordinated by the Department of Thoracic and Vascular Surgery. Tissues were maintained at 37°C for 6 h before inoculation with SFTSV or RVFV and harvested at 24 and 72 h post-infection (hpi) for subsequent analysis (Fig. 1A). Quantitative reverse transcription PCR (qRT-PCR) analysis revealed dose-dependent viral replication in the tissue (Fig. 1B through E). Viral RNA levels were higher at 72 hpi than at 24 hpi, consistent with progressive viral replication in the tissue explants. Immunoblotting further detected viral nucleoprotein in infected tissues, with greater NP abundance at 72 hpi than at 24 hpi for both viruses (Fig. 1F and G). To determine whether infection resulted in the production of infectious progeny viruses, we measured infectious virus released into the culture supernatants. Infectious SFTSV and RVFV were detected at 24 hpi, and supernatant viral titers further increased by 72 hpi (Fig. 1H). Together, these results demonstrate that *ex vivo* human vascular tissues support productive infection and infectious virus release by both SFTSV and RVFV.

### Bunyavirus infection triggers immune responses in the *ex vivo* vascular tissue

**Fig 2:**
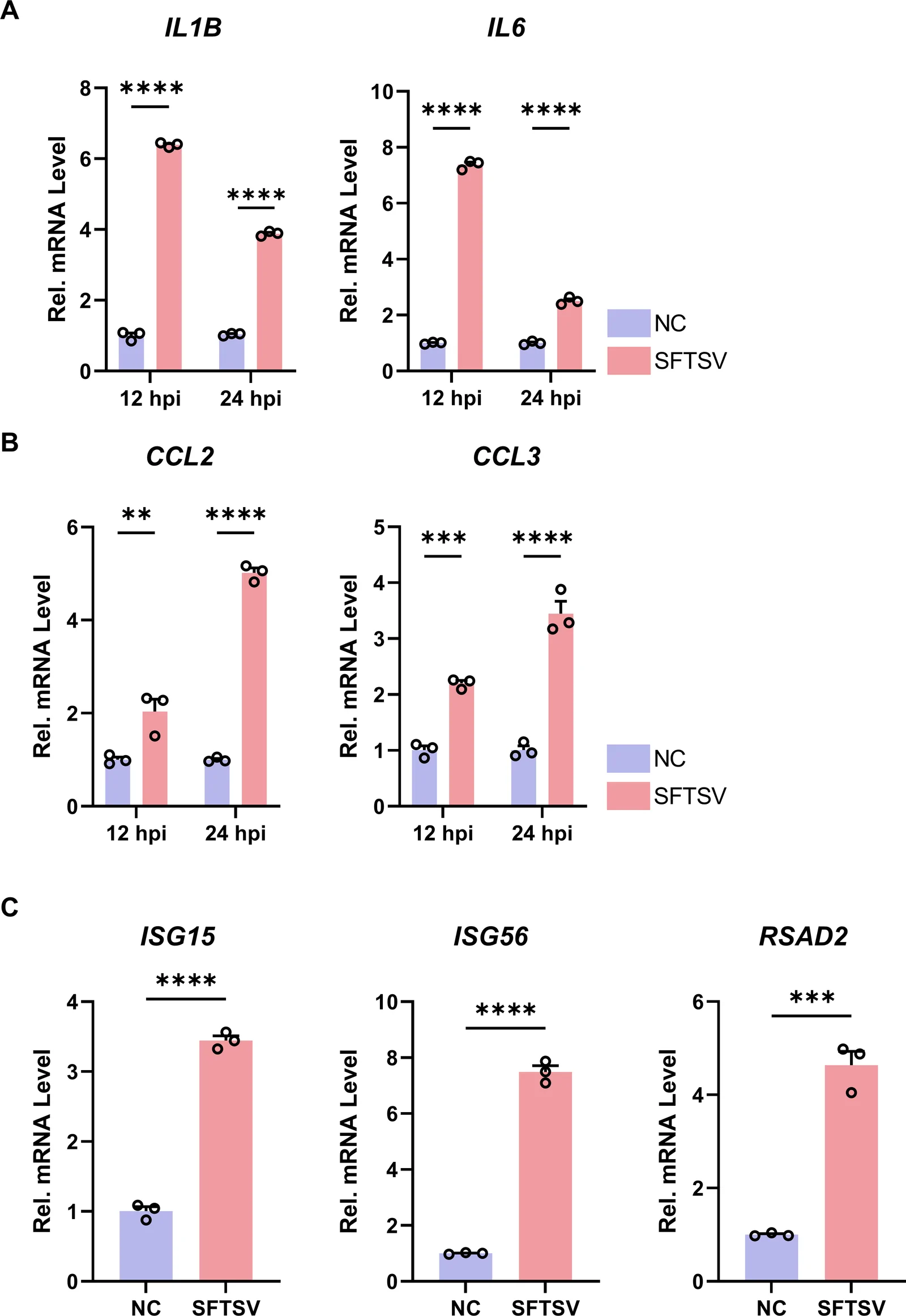
SFTSV infection triggers immune responses in *ex vivo* vascular tissue. (**A and B**) *Ex vivo* vascular tissues were inoculated with SFTSV at 4 × 10^6^ FFU per sample for 6 h, and infected tissues were collected at 12 and 24 hpi. Total RNA was extracted, and the relative mRNA levels of the proinflammatory cytokine genes *IL1B* and *IL6* (**A**) and the chemokine genes *CCL2* and *CCL3* (**B**) were detected by qRT-PCR. (**C**) *Ex vivo* vascular tissues were inoculated with SFTSV at 4 × 10^6^ FFU per sample for 6 h, and infected tissues were collected at 12 hpi. Total RNA was extracted, and the relative mRNA levels of the interferon-stimulated genes ISG15, ISG56, and RSAD2 were quantified by qRT-PCR. Data are presented as means ± SEM (*n* = 3, from three independent tissue donors) in panels **A–C**. Statistical significance was analyzed by two-way analysis of variance in panels **A** and **B** or Student’s *t*-test in panel **C**. **P < 0.01; ***P < 0.001; ****P < 0.0001.

Previous studies have demonstrated that both SFTSV and RVFV trigger inflammatory responses during infection (18, 19). Thus, we sought to investigate whether SFTSV and RVFV infections trigger innate immune responses in human *ex vivo* vascular tissue. To address this, vascular tissue was infected with SFTSV or RVFV, and the transcriptional expression of cytokine/chemokine genes and interferon-stimulated genes (ISGs) was analyzed. SFTSV infection induced upregulation of the proinflammatory cytokines interleukin-1β (*IL1B*) and interleukin-6 (*IL6*; Fig. 2A). Concurrently, elevated transcription levels of chemokines *CCL2* and *CCL3* were detected in SFTSV-infected vascular tissue (Fig. 2B). Notably, SFTSV infection markedly stimulated the expression of ISGs such as *ISG15*, *ISG56*, and *RSAD2* (Fig. 2C), indicating the activation of both inflammatory and antiviral transcriptional programs in the infected tissues. Similarly, RVFV infection induced upregulation of cytokine and chemokine genes in vascular tissue (Fig. S1A and B). To determine whether these responses required active infection rather than exposure to viral components alone, tissues were inoculated with live or heat-inactivated SFTSV and RVFV. Heat inactivation abolished detectable infectious virus production (Fig. S1C). Consistently, induction of cytokine, chemokine, and interferon-stimulated gene expression was substantially attenuated in tissues exposed to heat-inactivated viruses compared with tissues infected with the viruses (Fig. S1D). These results indicate that productive bunyavirus infection triggers a robust innate immune response in *ex vivo* human vascular tissue model.

### Bunyavirus infection induces endothelial damage in *ex vivo* vascular tissue

Prior to evaluating infection-associated pathological changes, we assessed the baseline stability of the *ex vivo* vascular tissue model over the experimental culture duration. In mock-cultured tissues, no progressive elevation in lactate dehydrogenase (LDH) release was detected from 24 to 72 h. Meanwhile, the expression levels of endothelial-associated genes, including *VWF*, *THBD*, *NOS3*, *ICAM1*, *VCAM1*, and *SELE*, showed no progressive decline during the culture period (Fig. S2A through C). These results confirm that the vascular tissue explants remained stable under the applied culture conditions.

**Fig 3:**
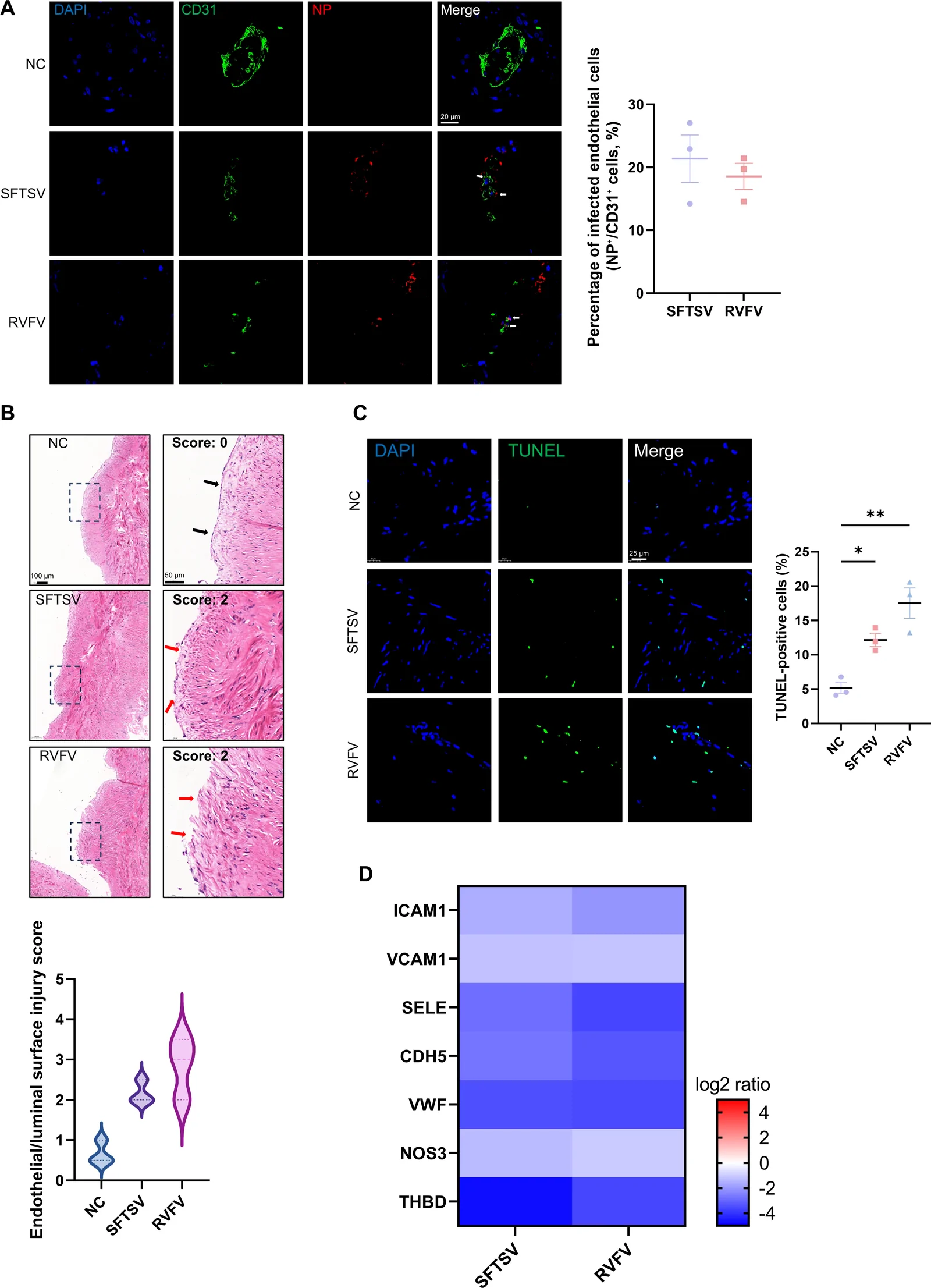
Bunyavirus infection induces endothelial injury and vascular dysfunction in *ex vivo* human vascular tissue. (**A**) *Ex vivo* vascular tissues were mock-infected (NC) or infected with SFTSV or RVFV (4 × 10^6^ FFU per sample) for 6 h. At 72 hpi, the tissues were fixed. Sections were co-stained with antibodies against viral NP (red) and CD31 (endothelial marker, green). Nuclei were stained with 4′,6-diamidino-2-phenylindole (DAPI; blue). Scale bar, 20 μm. Quantification of NP^+^/CD31^+^ endothelial cells is shown. (**B**) *Ex vivo* vascular tissues were mock-infected (NC) or infected with SFTSV or RVFV (4 × 10^6^ FFU per sample) for 6 h. At 72 hpi, the tissues were fixed and stained with hematoxylin and eosin (H&E). Black arrows indicate preserved luminal architecture, whereas red arrows indicate areas of endothelial surface disruption. Endothelial surface injury was evaluated using a blinded semiquantitative scoring system (0–4 scale). Scale bar, 100 μm; enlarged images, 50 μm. (**C**) *Ex vivo* vascular tissues were mock-infected (NC) or infected with SFTSV or RVFV (4 × 10^6^ FFU per sample) for 6 h. At 72 hpi, the tissues were fixed and stained with TUNEL (green) and DAPI (blue). Quantification of TUNEL-positive cells is shown. Scale bar, 25 μm. (**D**) *Ex vivo* vascular tissues were mock-infected (NC) or infected with SFTSV or RVFV (4 × 10^6^ FFU per sample) for 6 h. At 72 hpi, the tissues were harvested for total RNA extraction. Expression levels of endothelial barrier integrity and vascular function-associated genes were determined by qRT-PCR and normalized to 18S rRNA. The heatmap represents log2-transformed fold changes in gene expression relative to mock-treated tissues, with red and blue indicating increased and decreased expression, respectively. Data are presented as means ± SEM (*n* = 3, from three independent tissue donors) in panels **A and C**. Statistical significance was analyzed by one-way analysis of variance in panel **C**. *P < 0.05; **P < 0.01.

We next examined the cellular distribution of SFTSV and RVFV infection within the vascular tissues. Immunofluorescence co-staining revealed viral NP signals in CD31-positive endothelial cells along the luminal surface of infected tissues (Fig. 3A). Quantification of NP/CD31 double-positive cells confirmed that both viruses infected vascular endothelial cells. Co-staining analyses further revealed that viral NP was detected in h-Caldesmon-positive vascular smooth muscle cells and PDGFRα^+^/COL1A1^+^ fibroblast-associated populations (Fig. S3A and B).

Histopathological examination revealed virus infection-associated structural damage. Mock-infected control tissues maintained a continuous and regularly arranged luminal endothelial surface (score: 0). In contrast, SFTSV-infected tissues displayed focal irregularity and discontinuity of the endothelial lining with localized endothelial cell detachment, while RVFV-infected tissues exhibited more extensive luminal denudation and disruption of vascular wall organization (score: 2; Fig. 3B). Semiquantitative scoring of endothelial surface injury on a 0–4 scale confirmed increased injury scores following infection with either virus.

Consistent with these histopathological findings, TUNEL staining showed few positive cells in mock-infected control tissues, while a marked increase in TUNEL staining was observed following SFTSV or RVFV infection (Fig. 3C). Quantification of TUNEL-positive cells confirmed increased cell death in infected tissues. Infection also resulted in increased LDH release from the explants, further indicating impaired tissue integrity (Fig. S3C and D).

We further examined the expression of genes associated with endothelial identity, barrier integrity, and vascular homeostasis. At 72 hpi, both SFTSV and RVFV infections reduced the expression of multiple endothelial-associated genes, including *CDH5*, *VWF*, *THBD*, *NOS3*, *ICAM1*, *VCAM1*, and *SELE* (Fig. 3D and Fig. S3E and F). The coordinated downregulation of *CDH5*, *VWF*, *THBD*, and *NOS3* was consistent with disruption of endothelial barrier function and vascular homeostasis. In addition, altered expression of endothelial activation-associated genes, including *ICAM1*, *VCAM1*, and *SELE*, suggested dysregulation of endothelial inflammatory signaling following viral infection.

Together, these findings demonstrate that the *ex vivo* vascular tissue model remains relatively stable in the absence of infection, whereas SFTSV and RVFV infect vascular endothelial cells, resulting in structural disruption of the luminal surface, increased cell death, elevated LDH release, and altered expression of endothelial homeostasis-associated genes. These results indicate that bunyavirus infection triggers endothelial damage in this model.

### *Ex vivo* vascular tissue can be employed for evaluating antiviral compounds against bunyaviruses

The *ex vivo* vascular tissue model was susceptible to bunyavirus infection and exhibited pronounced pathological changes, making it a potential platform for antiviral drug evaluation. To validate this application, two chemical compounds with previously reported anti-SFTSV activities, niclosamide (Niclo) and 4′-fluorouridine (20, 21), were subjected to evaluation. The half-maximal inhibitory concentration (IC_50_) and 50% cytotoxic concentration (CC_50_) of these two compounds were determined in both Vero cells and human umbilical vein endothelial cells (HUVECs). As expected, both compounds exhibited potent antiviral activity with IC_50_ <1 µM and selectivity index (SI = CC_50_/IC_50_) >30 (Fig. S4A through D). Moreover, niclosamide and 4′-fluorouridine demonstrated dose-dependent inhibitory effects against RVFV, GTV, and HRTV in cell culture (Fig. S5A through F).

**Fig 4:**
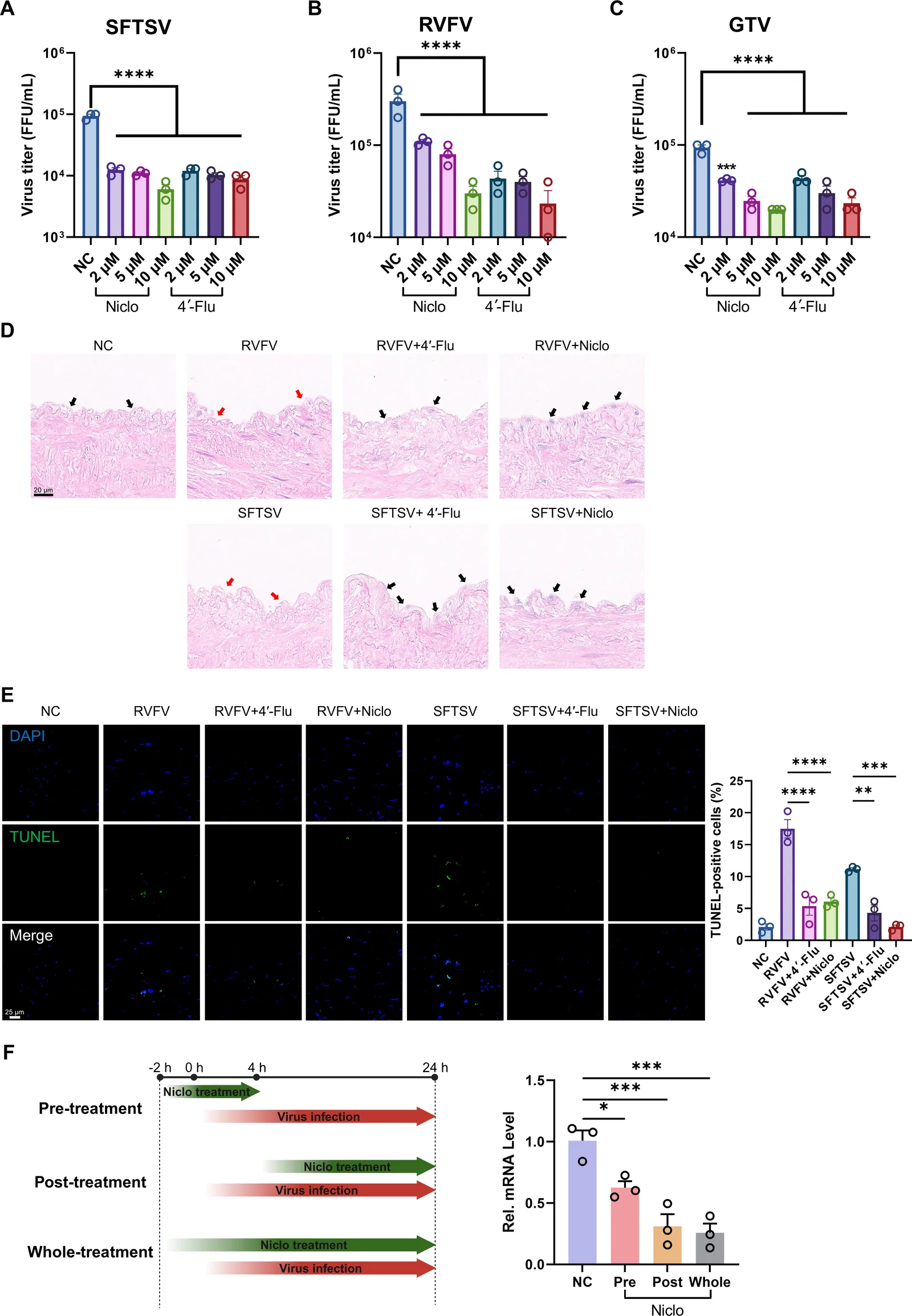
Niclosamide and 4′-fluorouridine suppress bunyavirus replication and reduce infection-associated vascular injury in *ex vivo* tissue. (**A-C**) *Ex vivo* human vascular tissues were infected with SFTSV (**A**), RVFV (**B**), or GTV (**C**) at 4 × 10^6^ FFU per sample. After a 6 h inoculation period, tissues were washed and subsequently treated with increasing concentrations of niclosamide or 4′-fluorouridine. Culture supernatants were collected at 24 hpi, and infectious virus production was quantified by focus-forming assay. Virus-specific vehicle-treated infected controls were included for each infection condition. (**D**) *Ex vivo* human vascular tissues were mock-infected (NC) or infected with SFTSV or RVFV (4 × 10^6^ FFU per sample). After a 6 h inoculation period, tissues were washed and subsequently treated with vehicle, niclosamide (10 μM), or 4′-fluorouridine (10 μM). At 72 hpi, the tissues were fixed for hematoxylin and eosin staining. Red arrows indicate areas of luminal surface disruption or endothelial injury, whereas black arrows indicate preserved vascular surface morphology. Scale bar, 20 μm. (**E**) *Ex vivo* human vascular tissues were mock-infected (NC) or infected with SFTSV or RVFV (4 × 10^6^ FFU per sample). After a 6 h inoculation period, tissues were washed and subsequently treated with vehicle, niclosamide (10 μM), or 4′-fluorouridine (10 μM). At 72 hpi, tissues were fixed and stained with TUNEL (green) and 4′,6-diamidino-2-phenylindole (DAPI; blue). Quantification of TUNEL-positive cells is shown. Scale bar, 25 μm. (**F**) Time-of-addition assay to determine the stage of SFTSV infection affected by niclosamide in *ex vivo* vascular tissues. Tissue explants were treated with niclosamide (10 μM) before infection (pre), after viral inoculation (post), or throughout infection (whole) as indicated. Viral RNA levels were quantified by qRT-PCR at 24 hpi. Data are presented as means ± SEM (*n* = 3, from three independent tissue donors) in panels **A–F**. Statistical significance was analyzed by one-way analysis of variance in panels **A–C**, **E**, and **F**. *P < 0.05; **P < 0.01; ***P < 0.001; ****P < 0.0001.

Since tissue sensitivity to pharmacological compounds may differ from that of immortalized cell lines, we assessed the toxicity of niclosamide and 4′-fluorouridine directly in *ex vivo* vascular tissues under the conditions used for antiviral evaluation using an ATP-based viability assay. Treatment with 4′-fluorouridine did not measurably reduce tissue viability across the tested concentrations. Niclosamide also showed tissue tolerability at the concentrations used for the antiviral assays, with a modest decrease in tissue viability observed at the highest concentration of 50 μM (Fig. S6A and B). qRT-PCR analysis showed that both niclosamide and 4′-fluorouridine reduced intracellular viral NP RNA levels in SFTSV-, RVFV-, and GTV-infected vascular tissues in a concentration-dependent manner (Fig. S6C through E). Consistent with the reduced viral RNA, infectious virus titration demonstrated significant reductions in SFTSV, RVFV, and GTV titers following treatment with either compound (Fig. 4A through C). Collectively, these results demonstrate that niclosamide and 4′-fluorouridine exhibit broad-spectrum antiviral activity against multiple bunyaviruses, with efficacy demonstrated in both cell culture systems and the *ex vivo* vascular tissue model.

To confirm that the compound concentrations selected for the tissue-protection analyses retained antiviral activity in the *ex vivo* vascular tissue model, we quantified infectious virus production under the same treatment conditions. Both niclosamide and 4′-fluorouridine significantly reduced infectious SFTSV and RVFV production from the tissue explants compared with the corresponding vehicle-treated infected controls (Fig. S6F). We then investigated whether suppression of viral replication was accompanied by attenuation of infection-associated vascular tissue injury. Histopathological examination of H&E-stained sections revealed that SFTSV- and RVFV-infected tissues displayed infection-associated vascular injury, including discontinuity of the endothelial lining, detachment of endothelial cells, and irregular luminal surfaces (red arrows; Fig. 4D). In contrast, these pathological alterations were markedly reduced in tissues treated with niclosamide or 4′-fluorouridine, which preserved a more continuous luminal surface architecture (black arrows; Fig. 4D).

Consistent with the histopathological findings, SFTSV and RVFV infections increased the proportion of TUNEL-positive cells, which was significantly reduced by niclosamide or 4′-fluorouridine treatment (Fig. 4E). Infection also increased LDH release from vascular tissue explants, indicative of impaired tissue integrity, which was attenuated by both compounds (Fig. S6G). We further evaluated the expression of genes associated with endothelial barrier integrity and vascular homeostasis. SFTSV and RVFV infections reduced the expression of *CDH5*, *VWF*, *THBD*, and *NOS3*, whereas treatment with niclosamide or 4′-fluorouridine partially restored their expression toward mock-treated levels (Fig. S6H). Together, these results show that the concentrations of niclosamide and 4′-fluorouridine used in the *ex vivo* tissue-protection experiments effectively suppressed viral RNA accumulation and infectious virus production, and that this antiviral activity was accompanied by reduced histopathological injury, cell death, LDH release, and partial restoration of endothelial homeostasis-associated gene expression.

To determine the stage of the SFTSV life cycle affected by niclosamide in the *ex vivo* tissue model, we next performed time-of-addition experiments. Niclosamide reduced viral NP RNA levels when administered before infection, after virus inoculation, or throughout the experimental period (Fig. 4F). These results suggest that niclosamide affects an early post-attachment step and subsequent stages of SFTSV infection. We further performed additional stage-specific assays in vascular tissues. Niclosamide treatment did not significantly alter the amount of tissue-associated SFTSV RNA under 4°C binding conditions, indicating that viral attachment was not affected (Fig. S7A and B). In contrast, after removal of unbound virions, niclosamide reduced internalized viral RNA during the entry phase in a concentration-dependent manner compared with vehicle-treated controls, supporting an inhibitory effect on viral internalization. When niclosamide was added after viral entry, both intracellular viral NP RNA levels and infectious virus titers were reduced, indicating additional antiviral activity at post-entry stages of infection (Fig. S7A through C). Collectively, these results demonstrate that *ex vivo* human vascular tissues provide a physiologically relevant platform for evaluating antiviral efficacy and virus-induced tissue injury.

### Niclosamide inhibits SFTSV infection by interfering with viral internalization, genome replication, and progeny virus release

**Fig 5:**
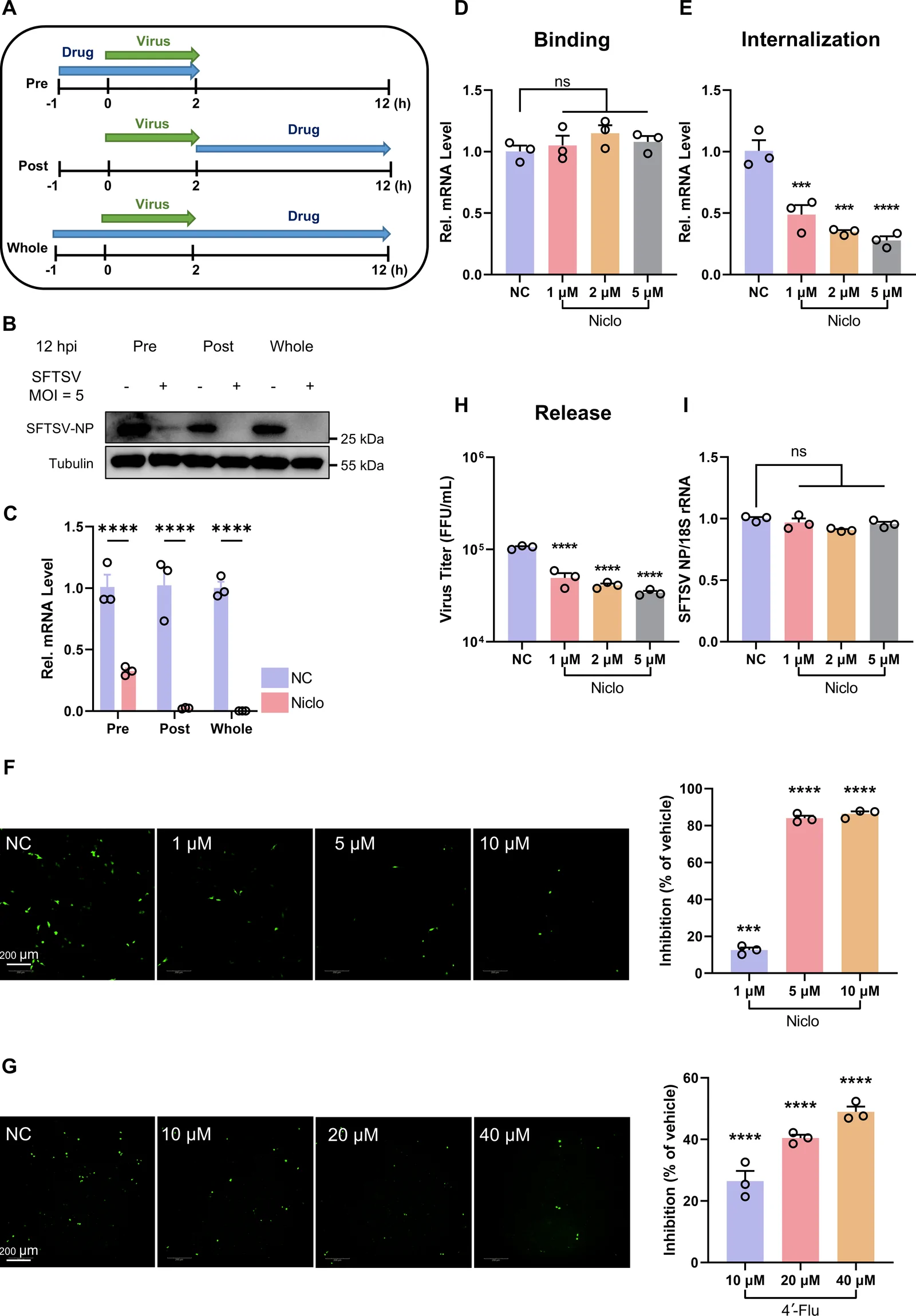
Niclosamide restricts multiple stages of the SFTSV life cycle. (**A**) Schematic diagram of the time-of-addition assay of niclosamide. (**B and C**) Vero cells infected with SFTSV at an MOI of 5 for 2 h were treated with niclosamide (2 μM) for 1 h pre-infection (pre), post-infection (post), and throughout the experiment (whole). Infected cells were collected at 12 hpi for western blot (**B**) and viral mRNA quantification (**C**). (**D**) Effect of niclosamide on SFTSV binding. Vero cells were pretreated with niclosamide or DMSO vehicle at 37°C for 1 h, chilled at 4°C for 15 min, then incubated with SFTSV at an MOI of 5 at 4°C for 2 h. Unbound virions were removed by washing three times with precooled PBS. Bound virions were then quantified by qRT-PCR. (**E**) Effect of niclosamide on SFTSV internalization. Vero cells pretreated with a series of concentrations of niclosamide for 1 h were chilled at 4°C for 15 min and incubated with SFTSV (MOI = 5) for 1 h. Cells were washed with precooled PBS to eliminate the unbound virions and incubated at 37°C for 3 h in the presence of ammonium chloride (20 mM), then washed and treated with trypsin. The relative level of internalized virions was determined by qRT-PCR. (**F and G**) Effect of niclosamide or 4′-fluorouridine on SFTSV MG activity. BSR-T7/5 cells were transfected with plasmids of the SFTSV MG system and treated with vehicle (DMSO or DMEM) or the indicated concentrations of niclosamide or 4′-fluorouridine. MG activity was assessed by quantification of the percentage of eGFP-positive cells. Scale bar, 200 μm. (**H and I**) Effect of niclosamide on SFTSV release. Vero cells were incubated with SFTSV (MOI = 5) at 37°C for 2 h, and then washed with PBS to remove the unbound virions. niclosamide was added at 10 hpi. At 14 hpi, the extracellular infectious progeny titers in the supernatant (**H**) and the corresponding intracellular viral mRNA levels (**I**) were simultaneously quantified. Data shown are means ± SEM (*n* = 3) from three independent experiments. Statistical significance was analyzed by one-way analysis of variance (ANOVA) in panels **D–I** or two-way ANOVA in panel **C**. ***P < 0.001; ****P < 0.0001; ns, not significant.

Time-of-addition assays in *ex vivo* vascular tissues indicated that niclosamide retained antiviral efficacy when administered after viral entry. Additional stage-specific analyses showed that niclosamide did not measurably affect SFTSV attachment but inhibited viral internalization (Fig. 4F and Fig. S7). Although the anti-SFTSV activity of niclosamide-containing formulations has been reported (20), the cellular mechanisms underlying niclosamide-mediated inhibition of SFTSV remain poorly understood. We therefore performed complementary cell-based assays to further investigate the antiviral mechanism of niclosamide. We first examined whether niclosamide directly inactivates SFTSV particles. SFTSV virions were incubated with niclosamide at the indicated concentrations for 1 h and subsequently diluted 10,000-fold to minimize residual compound activity before infectious titers were determined by focus-forming assay. The results indicated that niclosamide treatment did not significantly reduce viral infectivity, indicating the absence of a direct virucidal effect on SFTSV (Fig. S8A and B). Then, a time-of-addition assay was performed to identify the specific stage of the viral life cycle that was inhibited by niclosamide (Fig. 5A). Briefly, niclosamide was added to Vero cells before infection, after infection, or throughout SFTSV infection. The infected cells were harvested and subjected to qRT-PCR and western blot analysis to determine intracellular NP mRNA and protein levels. As shown in Fig. 5B and C, niclosamide treatment significantly reduced NP expression when administered either pre- or post-SFTSV infection, demonstrating its inhibitory effects during viral entry and replication stages.

Virion binding and internalization are two major steps during the viral entry stage. We next investigated whether SFTSV binding or internalization is targeted by niclosamide. For the binding inhibition assay, Vero cells pretreated with different concentrations of niclosamide or vehicle (DMSO) were cooled and incubated with SFTSV at 4°C for 2 h. Unbound virions were removed by washing three times with cold phosphate-buffered saline (PBS), and the cells were harvested for quantification of bound virions. As shown in Fig. 5D, the level of bound virions in niclosamide-treated groups was comparable to the control, suggesting that niclosamide did not affect the SFTSV binding step. To assess the effect of niclosamide on SFTSV internalization, Vero cells pretreated with different concentrations of niclosamide or vehicle were chilled at 4°C and incubated with SFTSV for 1 h. Then, the unbound virions were removed, followed by the addition of ammonium chloride to inhibit endosomal acidification and prevent subsequent replication. As shown in Fig. 5E, niclosamide treatment significantly reduced intracellular NP mRNA levels in a dose-dependent manner, indicating that niclosamide inhibited SFTSV internalization.

To investigate whether niclosamide specifically inhibits SFTSV genome replication, a mini-genome (MG) reporter assay was performed. Briefly, BSR-T7/5 cells were co-transfected with the mini-genome-eGFP plasmid and plasmids expressing viral L and NP proteins and treated with different concentrations of niclosamide. 4′-Fluorouridine, a potent inhibitor of viral genome replication, was used as a positive control. The eGFP signal was captured, and eGFP-positive cells were analyzed and quantified. As shown in Fig. 5F and G, treatment with both 4′-fluorouridine and niclosamide significantly reduced the percentage of eGFP-positive cells compared with the vehicle control. These results suggest that niclosamide inhibited SFTSV genome replication.

To further elucidate the antiviral mechanism of niclosamide, we investigated its effect on progeny virus release. For this purpose, cells were infected with SFTSV and treated with different concentrations of niclosamide at 10 hpi. At 14 hpi, the supernatant was harvested, and the infectivity of progeny virus was determined by a focus-forming assay. The results demonstrated that niclosamide treatment significantly reduced viral titer in a dose-dependent manner without substantially affecting intracellular viral RNA levels, suggesting that niclosamide reduced extracellular infectious virus production rather than merely reducing viral output as a consequence of impaired genome replication (Fig. 5H and I). Taken together, these results indicate that niclosamide inhibits SFTSV infection by interfering with viral internalization, genome replication, and progeny virus release.

### Niclosamide disrupts endolysosomal acidification and inhibits SFTSV internalization and infectious virus release

Since niclosamide has been reported to interfere with endolysosomal acidification and autophagic processing, we further investigated whether niclosamide affects autophagic flux during SFTSV infection. To assess the autophagic flux, we employed the tandemmCherry-eGFP-LC3B reporter system. In this system, LC3-positive structures located in nonacidified compartments retain both eGFP and mCherry fluorescence, whereas eGFP fluorescence is quenched after delivery into acidic autolysosomes. Thus, retention of the eGFP signal in mCherry-positive LC3 puncta is associated with defective compartment acidification and impaired late-stage autophagic flux.

**Fig 6:**
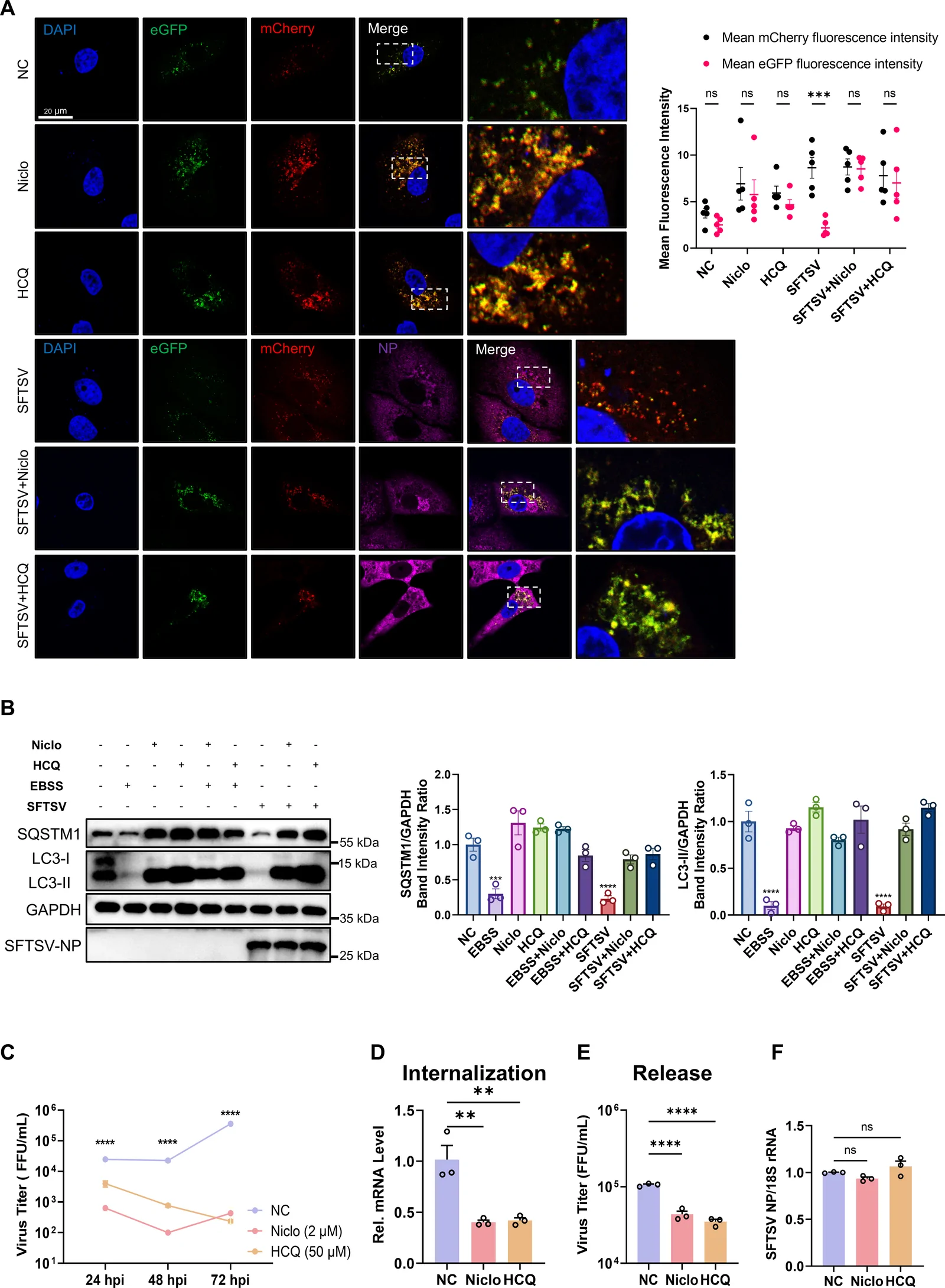
Niclosamide restricts SFTSV infection through disruption of acidification-dependent intracellular trafficking and autophagic flux. (**A**) The effect of niclosamide treatment on autophagic flux under SFTSV infection conditions was observed by confocal microscopy. Vero cells were transfected with the mCherry-eGFP-LC3B dual-fluorescence reporter plasmid for 24 h. Cells were infected with SFTSV (MOI = 5) for 18 h, followed by treatment with HCQ (50 μM) or niclosamide (1 μM) for 6 h. Representative confocal images show mCherry and eGFP signals in LC3-positive compartments. Increased eGFP retention and double-positive puncta indicate impaired acidification and defective late-stage autophagic flux. The scatter plot (right) shows the quantified mean fluorescence intensity of mCherry and eGFP. Scale bar, 20 μm. (**B**) The effect of niclosamide treatment on autophagic flux under SFTSV infection was analyzed by western blotting. Vero cells were subjected to starvation-induced autophagy by incubation in EBSS for 6 h, followed by treatment with HCQ (50 μM) or niclosamide (1 μM) for an additional 6 h. Alternatively, SFTSV-infected Vero cells were treated with HCQ (50 μM) or niclosamid (1 μM) at 18 h post-infection for 6 h. Cell lysates were immunoblotted for LC3 and SQSTM1/p62. Bar charts (right) display the densitometric quantification of LC3-II/GAPDH and SQSTM1/GAPDH band intensity ratio. (**C**) Effect of niclosamide (2 μM); HCQ (50 μM) on the production of infectious SFTSV progeny virions. Vero cells were treated with compounds and infected with SFTSV (MOI = 1), and progeny virions in the supernatant were collected and quantified at 24, 48, and 72 hpi. (**D**) Effect of niclosamide or HCQ on SFTSV internalization. Vero cells pretreated with niclosamide (2 μM) or HCQ (50 μM) for 1 h were chilled at 4°C for 15 min and incubated with SFTSV (MOI = 5) for 1 h. Cells were washed with precooled PBS to eliminate the unbound virions and incubated at 37°C for 3 h in the presence of ammonium chloride (20 mM), then washed and treated with trypsin. Cells were collected, and the relative level of internalized virions was determined by qRT-PCR. (**E and F**) Effect of niclosamide or HCQ on SFTSV release. Vero cells were infected with SFTSV (MOI = 5), and then niclosamide (2 μM) or HCQ (50 μM) was added at 10 hpi. At 14 hpi, the extracellular infectious progeny titers in the supernatant (**E**) and the corresponding intracellular viral mRNA levels (**F**) were simultaneously quantified. Data shown are means ± SEM (*n* = 3) from three independent experiments. Statistical significance was analyzed by two-way analysis of variance (ANOVA) in panels **A** and **C** or one-way ANOVA in panels **B** and **D–F**. **P < 0.01; ***P < 0.001; ****P < 0.0001; ns, not significant.

In SFTSV-infected cells, abundant mCherry-positive puncta accompanied by comparatively weak eGFP fluorescence were observed, consistent with the delivery of LC3-positive structures into acidic compartments. Niclosamide treatment preserved the eGFP signal and increased the accumulation of eGFP/mCherry double-positive puncta in SFTSV-infected cultures, producing a pattern comparable to that induced by hydroxychloroquine (HCQ), an inhibitor of lysosomal acidification (Fig. 6A). To exclude the possibility that the effects of HCQ resulted from overt cytotoxicity, we determined its 50% cytotoxic concentration in Vero cells. HCQ showed limited cytotoxicity at the concentrations used in autophagy and antiviral assays, with a calculated CC_50_ of approximately 139 μM in Vero cells (Fig. S9A). To determine whether this effect was independent of viral infection, we further examined autophagic flux under starvation-induced autophagy conditions. Niclosamide and HCQ similarly increased eGFP-positive LC3 puncta following Earle’s balanced salt solution (EBSS) stimulation (Fig. S9B), supporting a general effect of niclosamide on late-stage autophagic processing rather than a virus-specific artifact. These findings indicate that niclosamide impairs the acidification and/or turnover of LC3-positive compartments under both starvation and SFTSV infection conditions.

We next examined LC3 and SQSTM1/p62 turnover by immunoblotting. EBSS treatment reduced the steady-state abundance of LC3-II and SQSTM1/p62, consistent with increased autophagic turnover. Niclosamide treatment, similar to HCQ, resulted in accumulation of LC3-II and SQSTM1/p62 under both EBSS stimulation and SFTSV infection conditions (Fig. 6B). Densitometric analyses confirmed increased LC3-II/GAPDH and SQSTM1/p62/GAPDH ratios following niclosamide or HCQ treatment compared with the corresponding EBSS- or SFTSV-only controls. Together with the tandem-fluorescence reporter results, these data support the conclusion that niclosamide blocks late-stage autophagic flux during SFTSV infection.

Given the involvement of endolysosomal trafficking in multiple steps of viral infection, we next investigated whether the inhibition of autophagic/endolysosomal function affected distinct stages of SFTSV replication. Both compounds significantly reduced extracellular infectious virus titers throughout the 24- to 72-h infection period (Fig. 6C), accompanied by reductions in intracellular viral RNA levels (Fig. S9C). Stage-specific analysis further showed that treatment during the internalization period significantly decreased cell-associated SFTSV RNA, indicating impaired viral internalization (Fig. 6D).

To distinguish an effect on infectious-virus release from the consequence of reduced viral genome replication, niclosamide or HCQ was added during a restricted late-stage treatment window after viral replication. Under these conditions, both compounds modestly but significantly reduced extracellular infectious virus titers without significantly altering intracellular viral RNA levels in the corresponding samples (Fig. 6E and F). These results support an additional effect on the release of infectious progeny virus. These findings demonstrate that niclosamide disrupts late-stage autophagic flux and endolysosomal processing, resulting in inhibition of multiple stages of the SFTSV life cycle, including viral internalization and infectious progeny virus release.

We next examined whether the antiviral mechanisms identified in Vero cells could be recapitulated in HUVECs. HUVECs were treated with niclosamide or HCQ and subjected to stage-specific assays assessing viral internalization and late-stage infectious virus production. Similar to observations in Vero cells, both niclosamide and HCQ significantly reduced cell-associated SFTSV RNA following the internalization phase, suggesting reduced viral internalization in endothelial cells (Fig. S9D). To determine whether niclosamide also affects late-stage infectious virus production in endothelial cells, HUVECs were treated with niclosamide or HCQ after viral replication had been established. Under this restricted treatment window, both compounds significantly reduced extracellular infectious virus titers without substantially affecting intracellular viral RNA levels (Fig. S9E and F). These findings demonstrate that the stage-specific antiviral effects of niclosamide, including inhibition of viral internalization and reduction of infectious progeny virus production, are recapitulated in human endothelial cells.

## DISCUSSION

Bunyaviruses are arthropod-borne pathogens, some of which cause VHF with high fatality rates (22). The pathogenesis of VHFs involves severe vascular endothelial damage (16), and the mechanisms underlying endothelial dysfunction remain poorly elucidated. A major gap in current research is the absence of valid models that recapitulate vascular injury induced by virus infection. In this study, we demonstrated that the *ex vivo* vascular tissue could serve as a model for studying vascular endothelial injury caused by bunyavirus infection. Human vascular explants supported productive infection by SFTSV and RVFV and displayed infection-associated endothelial abnormalities, including endothelial infection, luminal surface disruption, increased cell death, and altered endothelial homeostasis-associated gene expression. Interestingly, SFTSV- and RVFV-infected tissues exhibited distinct histopathological patterns, suggesting virus-specific mechanisms of vascular injury. RVFV infection caused more extensive luminal disruption and tissue structural damage, consistent with its established association with severe vascular pathology and endothelial dysfunction. In contrast, SFTSV infection resulted in more localized endothelial alterations, which may reflect differences in viral replication kinetics, cellular tropism, and host inflammatory responses. These findings suggest that the *ex vivo* vascular tissue model can capture differential vascular responses induced by distinct bunyaviruses. In addition, infected tissues mounted coordinated inflammatory and antiviral transcriptional responses, demonstrating that this model preserves tissue-level innate immune responses that may be difficult to capture in conventional monoculture systems.

We acknowledge that the vascular tissues used in this study were obtained from patients with varicose veins, which may exhibit pre-existing endothelial dysfunction or a baseline inflammatory state compared to healthy vessels. Despite this limitation, the *ex vivo* tissues employed here retained the critical three-dimensional cytoarchitecture and native multicellular interactions that are absent in monolayer cell cultures, making them a more suitable system for evaluating the efficacy of antiviral compounds (23).

To assess whether the *ex vivo* vascular tissue model is suitable for the evaluation of antiviral compounds, we tested two compounds, niclosamide and 4′-fluorouridine, that have been reported to interfere with SFTSV infection (20, 21). Using this platform, we demonstrated their ability to suppress bunyavirus replication in human vascular tissues while reducing infection-associated tissue injury. niclosamide is a multifunctional compound previously reported to interfere with intracellular acidification and membrane trafficking pathways. Although niclosamide-containing formulations have shown anti-SFTSV activity (20), the inhibition mechanism of SFTSV remained incompletely defined. Our stage-specific assays demonstrated that niclosamide inhibits viral internalization and retains antiviral activity during post-entry stages. Niclosamide and 4′-fluorouridine also exhibited antiviral activity against multiple bunyaviruses, including SFTSV, RVFV, GTV, and HRTV. Future studies using vascular tissues from diverse donor populations and additional vascular-tropic pathogens will further define the applicability and limitations of this model.

Collectively, this study establishes an experimentally accessible human vascular tissue platform for investigating bunyavirus-associated vascular pathology. By integrating virological, histopathological, and molecular readouts, this model enables quantitative assessment of tissue injury and antiviral efficacy. Beyond SFTSV and RVFV, this approach may be applicable to other vascular-tropic pathogens and facilitate studies of virus-host interactions in a physiologically relevant tissue context.

## MATERIALS AND METHODS

### Cells and viruses

Vero cells and HUV-EC-C (HUVECs) were obtained from the American Type Culture Collection. BSR-T7/5 cells were provided by Dr. Mingzhou Chen (Hubei University, China). All cells were cultured in Dulbecco’s modified Eagle’s medium (DMEM; Gibco) supplemented with 10% fetal bovine serum (FBS; Gibco) and 1% antibiotics (Gibco). BSR-T7/5 cells were selected by the addition of 1 mg/mL G418 (Invitrogen) every other passage. The recombinant wild-type RVFV strain BJ01 was generated by reverse genetics as previously described (24). SFTSV strain HBMC16_human_2015 (25), GTV strain DXM (26), and HRTV (27) were obtained from the China Center for General Virus Culture Collection. All SFTSV and GTV infection experiments were performed in biosafety level 2 (BSL-2) facilities, whereas all RVFV and HRTV infection experiments were performed in biosafety level 3 (BSL-3) facilities, in compliance with standard institutional biosafety guidelines.

### Tissue culture and viral infection

Human vascular tissue samples were obtained from adult patients diagnosed with varicose veins. Vascular tissues obtained from patients were immediately rinsed with sterile normal saline and transported on ice. Tissue samples were sectioned into comparable-sized explants and placed into wells of 12-well plates containing 1 mL of complete culture medium, followed by incubation at 37°C for 6 h before viral inoculation. For infection, the culture medium was replaced with medium containing 2% FBS, and virus was added. At 6 hpi, tissues were washed three times with PBS and then maintained in 2% FBS medium until 24 or 72 hpi. Prior to sample collection, infected tissues were washed twice with PBS, lysed, and homogenized for subsequent western blotting or qRT-PCR analysis. For tissue-protection experiments, niclosamide or 4′-fluorouridine was added at the time of medium change after the 6 h viral inoculation period and maintained until harvest.

### Cell viability assay

Vero cells and HUVECs were seeded in 96-well plates and treated with different concentrations of drugs for 24 h. The cells were then incubated with Cell Counting Kit-8 (CCK-8; MedChemExpress) at 37°C for 1 h. Cell viability was measured according to the manufacturer’s instructions using a Synergy H1 microplate reader (BioTek). CC_50_ values were calculated by nonlinear regression using GraphPad Prism.

### *Ex vivo* tissue viability and LDH release assays

For *ex vivo* tissue viability assays, vascular tissue explants were cultured under mock conditions or treated with niclosamide or 4′-fluorouridine alone at the indicated concentrations for the same time window used for antiviral evaluation. Tissue viability was assessed using an ATP-based tissue viability assay according to the manufacturer’s instructions. To evaluate the baseline tissue integrity of the *ex vivo* model under mock-cultured conditions, culture supernatants were collected at 24, 48, and 72 h, and LDH release was measured according to the manufacturer’s instructions (Beyotime, #C0017).

### RNA isolation and quantitative reverse transcription PCR

Total RNA was extracted from infected cells or vascular tissues using VeZol Reagent (Vazyme, #R411) according to the manufacturer’s instructions. Tissue samples were homogenized prior to RNA extraction. qRT-PCR was performed using a two-step protocol. Viral RNA was reverse-transcribed into cDNA using the HiScript II 1st Strand cDNA Synthesis Kit (+gDNA wiper; Vazyme, #R212), followed by quantitative PCR using Taq Pro Universal SYBR qPCR Master Mix (Vazyme, #Q712) as previously described (28). The relative mRNA expression levels were calculated using the 2^−ΔΔCT^ method, with 18S rRNA serving as the endogenous control. Primer sequences are provided in Table S1.

### Western blot analysis

Cells and tissues were lysed in RIPA lysis buffer (P0013, Beyotime) supplemented with SDS-PAGE sample loading buffer. For western blot analysis, lysates from infected cells or tissues were separated by 12% SDS-PAGE and transferred onto a polyvinylidene difluoride membrane (Millipore, #ISEQ00010). The membranes were blocked with 5% (wt/vol) skim milk in TBS-T (0.2% Tween 20) and then incubated overnight at 4°C with the following primary antibodies: rabbit anti-viral nucleoprotein (NP antibody generated in-house), rabbit anti-LC3 (1:1,000 dilution; Cat No. 14600-1-AP; Proteintech), rabbit anti-SQSTM1 (1:1,000; Cat No. 18420-1-AP; Proteintech), rabbit anti-tubulin (1:3,000; Cat No. 11224-1-AP; Proteintech), and mouse anti-GAPDH (1:3,000; Cat No. 60004-1-Ig; Proteintech). After washing, membranes were incubated with HRP-conjugated secondary antibodies (1:5,000; anti-rabbit; Cat No. SA00001-2; anti-mouse; Cat No. SA00001-1-A; Proteintech). Protein bands were visualized using a ChemiScope 6000 imager (Clinx) with the SuperFemto ECL Chemiluminescence Kit (Vazyme, #E423).

### Immunofluorescence assay for autophagy detection

Vero cells were seeded onto coverslips and cultured until approximately 50% confluence. Cells were then transfected with the mCherry-eGFP-LC3B expression plasmid using Lipofectamine 3000 according to the manufacturer’s instructions. For starvation-induced autophagy assays, Vero cells were incubated in EBSS for 6 h to induce autophagy. Cells were then treated with niclosamide (1 μM) or HCQ (50 μM) in EBSS-containing medium for an additional 6 h before fixation or protein extraction. For SFTSV infection experiments, transfected cells were infected with SFTSV at an multiplicity of infection (MOI) of 5. At 18 hpi, cells were treated with niclosamide (1 μM) or HCQ (50 μM) for an additional 6 h. Following incubation, cells were washed with PBS and fixed with 4% paraformaldehyde for 30 min at room temperature. Then, cells were permeabilized with 0.3% Triton X-100 for 10 min, followed by blocking with 5% bovine serum albumin for 1 h. Nuclei were stained with 4′,6-diamidino-2-phenylindole (DAPI; Beyotime, #C1002) for 10 min. Finally, images were acquired using a confocal microscope (Leica STELLARIS 8 WLL). Autophagic flux was evaluated based on the differential stability of eGFP and mCherry fluorescence. Double-positive puncta represent LC3-positive compartments that have not undergone complete acidification, whereas mCherry-positive/eGFP-negative puncta indicate acidic autolysosomal compartments. The mean fluorescence intensity of mCherry and eGFP per unit area was quantified using Leica Application Suite X.

### Histology and immunohistochemistry

Infected and mock-treated *ex vivo* vascular tissue samples were fixed with 4% paraformaldehyde, embedded in paraffin, and cut into sections (4 μm). TUNEL staining was performed using the In Situ Cell Death Detection Kit (Roche, #11684817910) according to the manufacturer’s instructions.

For histology, vascular tissue samples were stained with Gill’s hematoxylin and eosin Y. Semiquantitative histopathological scoring was performed by an independent third-party pathology service provider with expertise in pathological evaluation. Evaluators were blinded to sample identities and experimental groups during the scoring process. The scoring criteria were defined as follows: 0, continuous intact endothelium; 1 , mild surface irregularity; 2, focal endothelial discontinuity or detachment; 3, extensive denudation; 4, severe diffuse structural disruption.

For immunofluorescence staining, sections were incubated with primary antibodies against viral nucleoprotein (NP antibody generated in-house) and cell-type markers, including CD31 (GB12064, Servicebio), h-Caldesmon (GB15960, Servicebio), PDGFRα (GB111342, Servicebio), and COL1A1 (GB155707, Servicebio) as indicated. Sections were then washed and incubated with appropriate Alexa Fluor-conjugated secondary antibodies, including Alexa Fluor 488 (GB25303, Servicebio), CY3 anti-rabbit (GB21303, Servicebio), and S-vision anti-rabbit (G1302, Servicebio). Nuclei were counterstained with DAPI. The sections were scanned using a DHISTECH Pannoramic MIDI scanner.

### Binding assay

To determine whether niclosamide affects SFTSV binding in the *ex vivo* vascular tissue model, vascular tissue explants were pretreated with niclosamide or vehicle control for 2 h and subsequently cooled to 4°C to restrict virus internalization. Tissues were then incubated with SFTSV at the indicated viral input at 4°C to allow viral attachment. After extensive washing with ice-cold PBS to remove unbound virions, tissues were collected and homogenized. Tissue-associated viral RNA was quantified by qRT-PCR as a measurement of bound virus.

To assess viral binding in the cell model, Vero cells were pretreated with niclosamide at the indicated concentrations or DMSO for 1 h at 37°C and chilled at 4°C for 15 min. Cells were then incubated with SFTSV (MOI = 5) at 4°C for 2 h to allow viral attachment. Unbound virions were removed by washing three times with precooled PBS. Cells were subsequently lysed using VeZol Reagent, and the relative amount of cell-bound viral RNA was quantified by qRT-PCR as previously described (28).

### Internalization assay

To evaluate the effect of niclosamide on viral internalization in vascular tissues, explants were pretreated with niclosamide or vehicle control and incubated with SFTSV under binding conditions at 4°C. Following removal of unbound virions by extensive washing, tissues were shifted to 37°C to permit internalization in the presence of niclosamide. Ammonium chloride (20 mM) was added to inhibit endosomal acidification and prevent subsequent viral replication. After incubation, tissues were washed and homogenized, and total RNA was extracted. Internalized viral RNA was quantified by qRT-PCR.

For viral internalization assays in a cell model, Vero cells were pretreated with different concentrations of niclosamide or vehicle (DMSO) for 1 h, chilled at 4°C for 15 min, and incubated with SFTSV (MOI = 5) for 1 h. Cells were washed to remove unbound virions and shifted to 37°C for 3 h in the presence of niclosamide and ammonium chloride (20 mM) to prevent endosomal acidification. Cells were then lysed with VeZol Reagent for total RNA extraction. The relative level of internalized viral RNA was determined by qRT-PCR as previously described (28).

### Post-entry replication assay in *ex vivo* tissue

To evaluate the effect of niclosamide on infectious progeny production from vascular tissues, infected tissue explants were treated with niclosamide or vehicle control after viral replication had been established. At the indicated time points, culture supernatants were collected and subjected to focus-forming assays to determine extracellular infectious virus titers. Corresponding tissue samples were collected for qRT-PCR analysis of intracellular viral RNA.

### Release assay in cell model

Vero cells were infected with SFTSV (MOI = 5) and incubated at 37°C for 2 h, and then washed three times with PBS to remove unbound virions. At 10 h post-infection, cells were treated with varying concentrations of niclosamide or vehicle (DMSO) for 4 h. Extracellular infectious virus titers in culture supernatants were determined by focus-forming assay. Simultaneously, the corresponding cells were lysed, and intracellular viral RNA levels were quantified by qRT-PCR.

### Mini-genome assay

The T7 mini-genome-eGFP reporter plasmid and expression plasmids encoding the L and NP viral proteins were co-transfected into BSR-T7/5 cells using Lipofectamine 3000 transfection reagent (Thermo Fisher Scientific). At 12 h post-transfection, cells were treated with different concentrations of niclosamide, 4′-fluorouridine, or DMSO vehicle control. The eGFP signal was imaged at 36 h post-transfection using the Operetta system (PerkinElmer) and analyzed using Harmony software version 3.5 (PerkinElmer).

### Statistical analyses

All data are presented as mean values ± SEM. For tissue experiments, *n* represents independent tissue donors; for cell culture experiments, *n* represents independent biological replicates. Statistical significance was determined with Student’s *t*-test, one-way analysis of variance (ANOVA), or two-way ANOVA, as indicated in the figure legends, with *P* < 0.05 considered statistically significant. ns, not significant; *P < 0.05; **P < 0.01; ***P < 0.001; ****P < 0.0001.

### Software

All statistical analyses were performed using GraphPad Prism version 10.1.0, as defined in the text and figure legends. Images were processed using Leica LAS X.

## Data Availability

All data are available in the main text or the supplemental material. Correspondence and requests for materials should be addressed to Ke Peng.

## SUPPLEMENTAL MATERIAL

Supplemental material mbio.01933-26-s0001.docx Supplemental figures and tables.

ASM does not own the copyrights to Supplemental Material that may be linked to, or accessed through, an article. The authors have granted ASM a non-exclusive, world-wide license to publish the Supplemental Material files. Please contact the corresponding author directly for reuse.
