# Supplementary material for: Establishment of an ex vivo vascular tissue model to investigate bunyavirus-triggered vascular endothelial injury
Source: mBio. 2026 Sep 25;17(10):e01933-26. doi: 10.1128/mbio.01933-26 (PMC13712801; doi:10.1128/mbio.01933-26)
Supplement: Supplementary file 1 [file mbio.01933-26-s0001.docx]

**Supplemental Material for:**

Establishment of an *ex vivo* vascular tissue model to investigate bunyavirus-triggered vascular endothelial injury

Zihan Guan^# a,b^, Xi Chen^# c^, Yujie Fang ^a,b^, Xiangtao Zhu ^a,b^, Shufen Li* ^a,b^, Ke Peng* ^a,b,d^

^a^ State Key Laboratory of Virology and Biosafety, Center for Antiviral Research, Center for Biosafety Mega-Science, Wuhan Institute of Virology, Chinese Academy of Sciences, Wuhan, Hubei, 430207, People’s Republic of China

^b^ University of Chinese Academy of Sciences, Beijing 101408, People’s Republic of China

^c^ Department of Vascular Surgery, Wuhan No. 1 Hospital, Wuhan, Hubei, 430022, People’s Republic of China

^d^ Lead contact

^#^ These authors contributed equally: Zihan Guan, Xi Chen

* Correspondence: lishufen@wh.iov.cn or pengke@wh.iov.cn.

**Figure S1. Productive bunyavirus infection induces innate immune activation in *ex vivo* human vascular tissues.**

(A and B) *Ex vivo* vascular tissue was inoculated with RVFV at 4 × 10⁶ FFU per sample for 6 h, and infected tissue was collected at 12 hpi. Total RNA was extracted, and the relative transcript levels of the indicated proinflammatory cytokines *IL1B* and *IL6* (A) and chemokines *CCL2* and *CCL3* (B) were determined by quantitative reverse transcription PCR (qRT-PCR).

(C) *Ex vivo* vascular tissues were inoculated with live or heat-inactivated SFTSV and RVFV at 4 × 10⁶ FFU per sample for 6 h. Culture supernatants were collected at 24 hpi, and infectious virus titers were determined by focus-forming assay. Representative focus-forming assay wells are shown on the right.

(D) *Ex vivo* vascular tissue explants were mock inoculated or inoculated with live or heat-inactivated SFTSV or RVFV at 4 × 10⁶ FFU per sample for 6 h and harvested at 24 hpi. The relative transcript levels of *CCL2*, *CCL3*, *IL1B*, *IL6*, *ISG15*, and *RSAD2* were quantified by qRT-PCR, normalized to 18S rRNA, and expressed relative to those in the corresponding mock-inoculated tissues.

Data are presented as means ± SEM (n = 3, from three independent tissue donors) in (A), (B), (C), and (D). Statistical significance was analyzed by Student’s t-test in (A) and (B). ***p* < 0.01; *****p* < 0.0001.

**Figure S2. Mock-cultured *ex vivo* vascular tissues maintain structural integrity during extended culture.**

(A) Culture supernatants were collected at 24, 48, and 72 h, and LDH release in supernatants was measured using the LDH Assay Kit.

(B and C) qRT-PCR analysis of endothelial homeostasis-associated genes in ex vivo vascular tissues at 24, 48, and 72 h.

Data are presented as means ± SEM (n = 3, from three independent tissue donors) in (A), (B), and (C). Statistical significance was analyzed by one-way ANOVA in (A), (B), and (C). ns, not significant.

**Figure S3. Cellular distribution of bunyavirus infection and endothelial dysfunction-associated responses in *ex vivo* human vascular tissues.**

(A) Detection of viral NP signals associated with vascular smooth muscle cell regions. Ex vivo vascular tissues were mock-infected (NC) or infected with SFTSV or RVFV (4 × 10⁶ FFU per sample) for 6 h. At 72 hpi, the tissues were fixed. Sections were co-stained with DAPI (nuclei, blue), h-Caldesmon (smooth muscle cell marker, cyan), and viral NP (red). Confocal images show NP-positive signals within h-Caldesmon-positive smooth muscle cell regions following SFTSV or RVFV infection. Scale bar, 20 μm. The percentage of NP⁺h-Caldesmon⁺ cells among total h-Caldesmon⁺ cells is shown.

(B) Detection of viral NP signals in fibroblast marker-positive stromal regions. Ex vivo vascular tissues were mock-infected (NC) or infected with SFTSV or RVFV (4 × 10⁶ FFU per sample) for 6 h. At 72 hpi, the tissues were fixed. Sections were co-stained with DAPI (blue), PDGFRα (fibroblast-associated marker, green), COL1A1 (extracellular matrix/fibroblast marker, cyan), and viral NP (red). Merged images demonstrate NP-positive signals within PDGFRα⁺/COL1A1⁺ regions in infected tissues. Scale bar, 20 μm. The percentage of NP⁺PDGFRα⁺COL1A1⁺ cells among total PDGFRα⁺COL1A1⁺ cells is shown.

(C and D) Quantification of infection-associated tissue damage and viral replication. Ex vivo vascular tissues were mock-infected (NC) or infected with SFTSV or RVFV (4 × 10⁶ FFU per sample) for 6 h. At 72 hpi, tissue injury was assessed by quantifying LDH release in culture supernatants (C). Viral replication was determined by measuring tissue-associated viral NP RNA levels by qRT-PCR (D). Viral RNA levels were normalized to 18S rRNA.

(E and F) Alteration of endothelial homeostasis-associated gene expression following bunyavirus infection. *Ex vivo* vascular tissues were mock-infected (NC) or infected with SFTSV or RVFV (4 × 10⁶ FFU per sample) for 6 h. At 72 hpi, the tissues were harvested for total RNA extraction. Expression levels of endothelial activation and junction-associated genes (*ICAM1*, *VCAM1*, *CDH5*, and *SELE*) (E) and vascular functional/coagulation-associated genes (*VWF*, *NOS3*, and *THBD*) (F) were quantified and normalized to 18S rRNA.

Data are presented as means ± SEM (n = 3 independent tissue donors) in (A), (B), (C), (D), (E), and (F). For immunofluorescence quantification, three randomly selected fields per donor were analyzed. Statistical significance was analyzed by one-way ANOVA in (C), (D), (E), and (F). **p* < 0.05; ***p* < 0.01; ****p* < 0.001; *****p* < 0.0001.

**Figure S4. Dose-response analysis of antiviral activity and cytotoxicity of Niclosamide and 4′-Fluorouridine.**

(A and C) Niclosamide and 4′-Fluorouridine exhibited dose-dependent inhibition of SFTSV replication in Vero cells. Vero cells were pretreated with a series of drug concentrations or DMSO or DMEM controls and then infected with SFTSV at an MOI of 1. At 24 hpi, cells were fixed and immunostained for nucleoprotein (NP) to analyze infected cells. Cell viability was measured by a CCK-8 assay. The inhibition rates and cell viability were normalized to DMSO or DMEM-treated cells. IC_50_ and CC_50_ values were calculated using GraphPad Prism 10.1.0. The chemical structure of each compound is displayed.

(B and D) Niclosamide and 4′-Fluorouridine exhibited dose-dependent inhibition of SFTSV replication in HUVECs. HUVECs were pretreated with a series of concentrations of the drugs or DMSO or DMEM and then infected with SFTSV at an MOI of 1. At 24 hpi, cells were fixed and immunostained for nucleoprotein (NP) to analyze infected cells. Cell viability was measured by a CCK-8 assay. The inhibition rates and cell viability were normalized to DMSO or DMEM-treated cells. IC_50_ and CC_50_ values were calculated using GraphPad Prism 10.1.0. The chemical structure of each compound is displayed.

For (A–D), data shown are means ± SEM (n = 3) from three independent experiments.

**Figure S5. Niclosamide and 4′-Fluorouridine have broad-spectrum anti-bunyavirus activity in vitro.**

(A–F) Vero cells pretreated with a series of concentrations of Niclosamide or 4′-Fluorouridine were infected with the bunyaviruses (HRTV, RVFV, and GTV) at an MOI of 1. At 24 hpi, intracellular RNA was extracted and viral RNA levels were measured by qRT-PCR.

Data shown are means ± SEM (n = 3) from three independent experiments. Statistical significance was analyzed by one-way ANOVA. *****p* < 0.0001.

**Figure S6. Niclosamide and 4′-Fluorouridine inhibit multiple bunyaviruses and attenuate infection-associated injury in *ex vivo* human vascular tissues.**

(A and B) Effect of 4′-Fluorouridine and Niclosamide on *ex vivo* human vascular tissue viability. Uninfected ex vivo vascular tissues were treated with increasing concentrations (1.5625 to 50 μM) of 4′-Fluorouridine (A) or Niclosamide (B), corresponding to the concentrations used for antiviral evaluation. Tissue viability was assessed using an ATP-based viability assay and expressed as a percentage of vehicle-treated control tissues, which were set to 100%.

(C–E) Dose-dependent broad-spectrum inhibition in *ex vivo* vascular tissues. *Ex vivo* human vascular tissues were infected with SFTSV (C), RVFV (D), or GTV (E) at 4 × 10⁶ FFU per sample. After a 6 h inoculation period, tissues were washed and subsequently treated with increasing concentrations of Niclosamide or 4′-Fluorouridine. At 24 hpi, total RNA was extracted from the homogenized tissues, and the relative intra-tissue mRNA levels of viral nucleoprotein (NP) were quantified by qRT-PCR and normalized to 18S rRNA.

(F–H) Antiviral activity and attenuation of infection-associated tissue injury at the concentrations selected for tissue-protection analyses. *Ex vivo* human vascular tissues were mock-infected (NC) or infected with SFTSV or RVFV (4 × 10⁶ FFU per sample), followed by treatment with vehicle, Niclosamide (10 μM), or 4′-Fluorouridine (10 μM). Parallel tissue explants from the same donors were used for viral titration (F), LDH measurement (G), and qRT-PCR analyses (H).

(F) The titers of infectious progeny virions released into the tissue culture supernatants were determined by focus-forming assay.

(G) Lactate dehydrogenase (LDH) release into the culture supernatants was measured as an indicator of loss of tissue cellular integrity.

(H) The transcript levels of the endothelial barrier- and homeostasis-associated genes *CDH5*, *VWF*, *THBD*, and *NOS3* were quantified by qRT-PCR, normalized to 18S rRNA, and expressed relative to the vehicle-treated mock-inoculated control.

Data are presented as means ± SEM (n = 3, from three independent tissue donors) in (A), (B), (C), (D), (E), (F), (G), and (H). Statistical significance was analyzed by one-way ANOVA in (C), (D), and (E) and by two-way ANOVA in (F), (G), and (H). **p* < 0.05; ***p* < 0.01; ****p* < 0.001; *****p* < 0.0001.

**Figure S7. Stage-specific antiviral activity of Niclosamide in *ex vivo* human vascular tissues.**

(A) Schematic representation of the stage-specific experimental workflows employed in the *ex vivo* vascular tissue model. In the binding assay, tissues were pretreated with Niclosamide (1 μM or 10 μM) at 37°C for 2 h, followed by incubation with SFTSV (4 × 10⁶ FFU per sample) at 4°C for 4 h to allow viral attachment while restricting endocytosis. In the internalization assay, pretreated tissues were chilled at 4°C for 2 h to permit viral binding, then shifted to 37°C for 6 h to trigger viral internalization, with 20 mM NH₄Cl added at 2 hpi to prevent subsequent rounds of infection. In the post-entry replication assay, tissues were infected with SFTSV for 12 h prior to the addition of Niclosamide, isolating the drug’s inhibitory effect on late-stage viral replication events.

(B) Effect of Niclosamide on SFTSV binding and internalization in vascular tissues. Following the protocols in (A), tissues were homogenized, total RNA was extracted, and the relative levels of intra-tissue viral RNA (NP mRNA) were quantified by qRT-PCR.

(C) Effect of Niclosamide on post-entry viral replication in vascular tissues. Tissues were treated with Niclosamide during the restricted late-stage window (12–24 hpi). Intra-tissue viral mRNA levels were quantified by qRT-PCR (left). The titers of infectious progeny virions released into the culture supernatant were determined by focus-forming assay (right).

Data are presented as means ± SEM (n = 3, from three independent tissue donors) in (B) and (C). Statistical significance was analyzed by one-way ANOVA in (B) and (C). **p* < 0.05; ***p* < 0.01; ns, not significant.

**Figure S8. Virucidal activity of Niclosamide and schematics of stage-specific antiviral assays.**

(A) Schematic diagram of the virucidal assay.

(B) Virucidal effect of Niclosamide on SFTSV. SFTSV was preincubated with different concentrations of Niclosamide or DMSO for 1 h at 22°C. The mixture was diluted 10,000-fold and subjected to viral titer determination.

(C) Schematic diagram of the binding, internalization, and release assays.

Data shown are means ± SEM (n = 3) from three independent experiments. Statistical significance was analyzed by one-way ANOVA. ns, not significant.

**Figure S9. HCQ-mediated inhibition of autophagic flux restricts SFTSV infection and** **HUVEC-based** **validation of Niclosamide stage-specific antiviral activity.**

(A) Cytotoxicity of Hydroxychloroquine (HCQ) in Vero cells. Cell viability was measured by a CCK-8 assay after 24 h of treatment with varying concentrations of HCQ, and the half-maximal cytotoxic concentration (CC_50_) was calculated to ensure subsequent mechanism assays were performed at non-toxic doses.

(B) Niclosamide impairs autophagic flux under starvation conditions. Vero cells were transfected with the tandem fluorescent autophagy reporter plasmid mCherry-eGFP-LC3B for 24 h. Cells were then starved in Earle's Balanced Salt Solution (EBSS) to induce autophagy, followed by treatment with HCQ (50 μM) or Niclosamide (1 μM) in EBSS medium for an additional 6 h. Representative confocal images display the accumulation of mCherry/eGFP double-positive LC3 puncta, indicating impaired acidification. The scatter plot (below) shows the quantified mean fluorescence intensity of mCherry and eGFP. Scale bar, 20 μm.

(C) Effect of Niclosamide or HCQ on intracellular mRNA levels of SFTSV NP. Vero cells were treated with Niclosamide (2 μM) or HCQ (50 μM) and infected with SFTSV (MOI = 1). Intracellular viral RNA was extracted at 24, 48, and 72 hpi, and the relative mRNA level of SFTSV NP was quantified by qRT-PCR.

(D) Effect of Niclosamide or HCQ on SFTSV internalization. HUVECs pretreated with Niclosamide (2 μM) or HCQ (50 μM) for 1 h were chilled at 4°C for 15 min and incubated with SFTSV (MOI = 5) for 1 h. Cells were washed with precooled PBS to eliminate unbound virions and incubated at 37°C for 3 h in the presence of ammonium chloride (20 mM), and then washed and treated with trypsin. Cells were collected, and the relative level of internalized virions was determined by qRT-PCR.

(E and F) Effect of Niclosamide or HCQ on SFTSV release. HUVECs were infected with SFTSV (MOI = 5) and then Niclosamide (2 μM) or HCQ (50 μM) was added at 10 hpi. At 14 hpi, the extracellular infectious progeny titers in the supernatant (E) and the corresponding intracellular viral mRNA levels (F) were simultaneously quantified.

Data shown are means ± SEM (n = 3) from three independent experiments. Statistical significance was analyzed by two-way ANOVA in (B) and (C) or one-way ANOVA in (D), (E), and (F). ***p* < 0.01; ****p* < 0.001; *****p* < 0.0001; ns, not significant.

**Table S1. Primers used for qRT-PCR.**

| **Gene target** | **Direction** | **Primer sequence (5′→3′)** |
| --- | --- | --- |
| 18S rRNA | Forward | TGAGAAACGGCTACCACATC |
|  | Reverse | TTACAGGGCCTCGAAAGAGT |
| ISG15 | Forward | CTGTTCTGGCTGACCTTCG |
|  | Reverse | GGCTTGAGGCCGTACTCC |
| ISG56 | Forward | TCAGGTCAAGGATAGTCTGGAG |
|  | Reverse | AGGTTGTGTATTCCCACACTGTA |
| RSAD2 | Forward | TGCCTGAATCTAACCAGAAGATGA |
|  | Reverse | GGATGGACTTGGAAGGGTCC |
| CCL2 | Forward | GCCAGATGCAATCAATGCCC |
|  | Reverse | GGTTTGCTTGTCCAGGTGGT |
| CCL3 | Forward | ATTCCGTCACCTGCTCAGAA |
|  | Reverse | TGGCTGCTCGTCTCAAAGTA |
| IL1B | Forward | CACGATGCACCTGTACGATCA |
|  | Reverse | GTTGCTCCATATCCTGTCCCT |
| IL6 | Forward | AGACAGCCACTCACCTCTTCAG |
|  | Reverse | TTCTGCCAGTGCCTCTTTGCTG |
| SFTSV-NP | Forward | ATGTCAGAGTGGTCCAGGA |
|  | Reverse | TCTCCACCTGTCTCCTTCAG |
| GTV-NP | Forward | TAAAGAGGTGGGAGGTGA |
|  | Reverse | CATTAGGGAAGAAGACAGAG |
| RVFV-NP | Forward | TAAGGGCGATATTGGATGCT |
|  | Reverse | TTGCAGCAACTTCCTCCTTT |
| HRTV-NP | Forward | CCTTTGGTCCACATTGATTG |
|  | Reverse | CACTGATTCCACAGGCAGAT |
| VCAM1 | Forward | GGGTCTACCAGCTCCAGAGA |
|  | Reverse | GGGATTCACAGCCCATGACA |
| ICAM1 | Forward | AGGATGGCACTTTCCCACTG |
|  | Reverse | TCCCTTTTTGGGCCTGTTGT |
| E-selectin (SELE) | Forward | GGCAGTTCCGGGAAAGATCA |
|  | Reverse | AGCCAGAGGAGAAATGGTGC |
| CDH5 | Forward | CGACCTCCGATACATGAGCC |
|  | Reverse | TGTGACTCGGAAGAACTGGC |
| NOS3 | Forward | TAACACAGACAGTGCAGGGG |
|  | Reverse | CTCCGTTTGGGGCTGAAGAT |
| THBD | Forward | ACATCCTGGACGACGGTTTC |
|  | Reverse | AGATGCCTATGAGCAAGCCC |
| VWF | Forward | GAGCTGCGAGGAGAGGAATC |
|  | Reverse | AGTCTTCAGGGTCAACGCAG |
